# Bleeding Risk of Anticoagulation Reversal Strategies Before Heart Transplantation: A Retrospective Comparative Cohort Study

**DOI:** 10.3390/jcdd11110366

**Published:** 2024-11-13

**Authors:** Antonio Prieto-Romero, Sara Ibañez-García, Xandra García-González, Javier Castrodeza, Beatriz Torroba-Sanz, Carlos Ortiz-Bautista, Cristina Pascual-Izquierdo, José María Barrio-Gutiérrez, Ángel González-Pinto, Ana Herranz-Alonso, María Sanjurjo-Sáez

**Affiliations:** 1 Department of Pharmacy, Hospital General Universitario Gregorio Marañón, Instituto de Investigación Sanitaria Gregorio Marañón, 28007 Madrid, Spain; antonio.prieto@salud.madrid.org (A.P.-R.); xandra.garcia@salud.madrid.org (X.G.-G.); beatriz.torroba@salud.madrid.org (B.T.-S.); aherranza@salud.madrid.org (A.H.-A.); maria.sanjurjo@salud.madrid.org (M.S.-S.); 2 Department of Cardiology, Hospital General Universitario Gregorio Marañón, CIBER de Enfermedades Cardiovasculares (CIBERV), 28007 Madrid, Spain; javier.castrodeza@salud.madrid.org (J.C.); carlos.ortiz@salud.madrid.org (C.O.-B.); 3 Department of Hematology, Hospital General Universitario Gregorio Marañón, Instituto de Investigación Sanitaria Gregorio Marañón, 28007 Madrid, Spain; cpascuali@salud.madrid.org; 4 Department of Anaesthesia, Hospital General Universitario Gregorio Marañón, Instituto de Investigación Sanitaria Gregorio Marañón, 28007 Madrid, Spain; jbarriog@salud.madrid.org; 5 Department of Cardiac Surgery, Hospital General Universitario Gregorio Marañón, Instituto de Investigación Sanitaria Gregorio Marañón, 28007 Madrid, Spain; agpinto@salud.madrid.org

**Keywords:** idarucizumab, anticoagulation reversal, bleeding risk, heart transplantation, economic analysis

## Abstract

Heart transplantation (HT) poses high bleeding risks, especially for patients on anticoagulation. This study evaluates the use of idarucizumab for dabigatran (DBG) reversal compared to vitamin K antagonist (VKA) strategies in HT. A retrospective analysis of HT patients from January 2018 to December 2022, excluding those requiring ECMO immediately before or after surgery, was conducted. Outcomes included transfusion needs, re-surgery due to bleeding, ICU stay lengths, and 30-day survival. A cost analysis compared the direct expenses of each strategy. Among 34 patients, 20 were on DBG and 14 on VKAs or not anticoagulated. Idarucizumab significantly reduced the number of patients requiring transfusion (*p* = 0.034) and ICU stay lengths (*p* = 0.014), with no significant impact on re-surgery rates (*p* = 0.259) or survival (*p* = 0.955). Despite higher initial costs, overall expenses for idarucizumab were comparable to VKA reversal due to reduced transfusion needs and shorter ICU stays. Idarucizumab offers a viable and potentially cost-neutral anticoagulation reversal option for HT patients on DBG, presenting an alternative to VKA strategies. However, due to the retrospective nature of the study and the small sample size, further prospective studies are needed to confirm these findings.

## 1. Introduction

Heart transplantation (HT) is a major surgical procedure with high hospital mortality related largely to the high incidence of hemorrhagic complications. Many patients on HT waiting lists require anticoagulation, which further increases this risk [1], making its preoperative reversal a key aspect to ensure the safety of the surgery.

The reversal of vitamin K antagonists (VKAs) can be rapidly achieved upon the availability of a donor’s heart with the administration of vitamin K and prothrombin complex concentrates (PCCs). Regarding direct oral anticoagulants (DOACs), the lack of an antidote has precluded their use until the authorization of idarucizumab in October 2015, a monoclonal antibody fragment developed to reverse specifically the anticoagulant effect of dabigatran (DBG). Despite the scarce evidence supporting the use of idarucizumab in the HT setting, it suggests it is safe and effective [2]. However, we have not found any study comparing its efficacy, safety, and economic viability with the reversal of VKAs. We consider that addressing this gap is key for selecting the most appropriate anticoagulant therapy for HT candidates, especially considering that DOACs confer advantages over VKAs in terms of efficacy and safety [3,4].

Drawing from our center’s clinical experience, we hypothesized that employing an idarucizumab reversal strategy could be linked to improved clinical outcomes, and we conducted this study to evaluate it.

## 2. Materials and Methods

Study design, objectives, patient selection, and ethical considerations

- This was a retrospective cohort study conducted at a tertiary Spanish hospital.
- Primary objective: to compare the effectiveness and safety of using idarucizumab (Praxbind^®^ 2g/50 mL, Boehringer Ingelheim International), for the reversal of DBG with that of reversing VKAs before HT surgery, based on the need of transfusion of blood derivatives, re-surgery after HT due to bleeding, length of ICU stay, or 30-day survival.
- Secondary objective: to conduct a cost analysis of both anticoagulant reversal strategies focusing on the direct expenses associated with the use of anticoagulation reversal drugs, blood product transfusions, and the length of ICU stay.
- Inclusion criteria: Patients over 18 years who received HT in our center from January 2018 to December 2022. These patients were classified according to whether they were taking DBG or VKAs, or were not anticoagulated (NA) at the time of their HT. The NA group was analyzed to understand the baseline transfusion requirements of non-anticoagulated patients.
- Exclusion criteria: patients who were given extracorporeal membrane oxygenation (ECMO) before the HT or during the immediate postoperative period, defined as the first 48 h post-surgery, due to a higher coagulopathy risk.
- The study was approved by the Institutional Review Board with a waiver of informed consent (IRB, TC-ACO-2024).

Local Protocols for Anticoagulation Reversal and Transfusions

- To reverse DBG, idarucizumab was administered as two 2.5 g boluses within 15 min of each other, regardless of the aPTT value, DBG dose, timing of the last DBG administration, or the baseline estimated glomerular filtration rate (eGFR). VKA reversal was achieved by administering 10 mg of vitamin K along with 4F-PCC dosed as 500 IU for an international normalized ratio (INR) range of 1.5–2, 1000 IU for an INR of 2–2.5, and 1500 IU for an INR of 2.5–3, while weight-based dosing was used if the INR was above 3, according to the manufacturer’s instructions.
- Our local transfusion protocol indicates using a restrictive packed red blood cell (PRBC) transfusion strategy, targeting Hb levels within the range of 7–8 g/dL. For pool of platelets (POP) and fresh frozen plasma (FFP), we adhered to the rotational thromboelastometry (ROTEM) A5 algorithm for cardiovascular surgery, as outlined by Görlinger et al. [5] and the 2019 Society of Cardiovascular Anesthesiologists ROTEM, and a thromboelastography (TEG)-based cardiac surgery intraoperative transfusion algorithm [6].

Variables and outcomes

- Population variables: Data were collected from the Hospital’s electronic medical records and prescribing system. Variables included the age at the time of HT, sex, etiology of heart disease, anticoagulant indication and regimen, CHA2DS2-VASc scores, reversal agents and their doses, and pre-surgery biochemical data, including renal function assessed by the Modification of Diet in Renal Disease eGFR formula. Information on hemostasis was also collected, including the preoperative and postoperative INR and activated partial thromboplastin time (aPTT), to evaluate the effects of VKA and DBG reversal, respectively. To evaluate basal bleeding risk, we analyzed the history of previous sternotomies—a recognized risk factor for increased surgical bleeding in heart surgery—concomitant antiplatelet use, CPB (cardiopulmonary bypass) time, the occurrence of simultaneous organ transplant procedures, and the HAS-BLED score, which assesses the risk of bleeding in patients on anticoagulation therapy based on factors such as hypertension, abnormal renal or liver function, stroke history, prior bleeding, labile INR, age, and drug/alcohol use. Finally, we recorded whether the HT occurred during the course of an index admission, identifying patients who were likely more unstable and thus may be at a higher risk of bleeding complications.
- Clinical outcome variables: Blood products were used as surrogate measures for bleeding in the immediate postoperative period (up to 48 h post-surgery), so data were collected on the use of PRBCs, FFP, and POP. Additionally, the necessity for re-surgery due to bleeding complications, length of ICU stay, and 30-day survival post-HT were evaluated. Our evaluation prioritized the length of ICU stay over total admission duration to more precisely assess bleeding during and after surgery. Patients who died during surgery due to massive bleeding were counted as transfused to accurately capture significant bleeding events as a clinical outcome. However, these patients were then excluded from the remaining postoperative outcome analyses (e.g., total blood products transfused, length of ICU stay, and re-surgery due to bleeding) to avoid distorting the results.
- Cost analysis variables: We compared the costs incurred by the DBG and VKA cohorts, collecting direct medical costs from the reversal agents, the transfusion of blood products, and ICU stay length. Cost data, collected at the time of manuscript preparation, were based on the maximum ex-factory prices, inclusive of VAT. The average daily cost of an ICU stay was sourced from the accounting department of our hospital, while the cost of blood products was derived from our hematology catalog. These cost details are outlined in Table 1. A sensitivity analysis was conducted to evaluate the impact of ICU stay costs, exploring the best- and worst-case scenarios by analyzing the extreme variations in ICU costs, using the lower and upper bounds of the confidence intervals.

Statistical methods

Data analysis was performed using Stata statistical software (version 17, StataCorp, College Station, TX, USA). For non-parametric data comparisons, we employed Kruskal–Wallis tests followed by post hoc Dunn tests. Pearson’s chi-square tests were used for binary categorical variables. A Holm–Bonferroni correction was applied for multiple comparison adjustment, obtaining adjusted *p*-values that were considered statistically significant when lower than 0.05. For the cost analysis, Mann–Whitney U tests were performed, while the Hodges–Lehmann estimator was used to obtain confidence intervals (CIs) for the sensitivity analysis.

## 3. Results

### 3.1. Patient Characteristics

During the research period, our hospital performed a total of 58 HTs. Of these, 10 patients were excluded from the study due to the utilization of an ECMO device before the HT or during the immediate postoperative period. The remaining 48 eligible patients comprised 20 patients on DBG, 14 on VKAs (13 on acenocoumarol and one on warfarin), and 14 NA.

Patient characteristics are detailed in Table 2. In the study, patient ages were broadly similar across the DBG, VKA, and NA cohorts. Both the DBG and VKA cohorts’ HAS-BLED score was 0. Most patients on VKAs had INR targets of 2–3, except for two patients with mechanical mitral valves, who had targets of 2.5–3.5. These two patients also had a previous sternotomy for their valve replacement.

The NA group showed more high-risk bleeding features, with a greater prevalence of previous sternotomy (42.9%) compared to the VKA group (21.4%) and the DBG group (15.8%), a more common use of anti-aggregation therapy (46.2% in the NA group vs. 14.3% in the VKA group and none in the DBG group), and a higher rate of HT during ongoing admissions (42.9% in the NA group, none in the VKA group, and 5% in the DBG group). While some patients in the DBG and VKA groups had either re-sternotomy or anti-aggregation therapy, none had both factors concurrently. Furthermore, while DBG patients exclusively had heart-only transplants, the VKA and NA groups had more complex cases, including two combined transplants (cardiorenal and cardiohepatic) in the VKA group and one cardiohepatic in the NA group. Differences in the cardiopulmonary bypass (CPB) time were not statistically significant (*p* = 0.080, Table 3).

### 3.2. Clinical Outcomes

In the DBG cohort, median aPTT decreased from a median of 47.4 s preoperatively to 28.2 s postoperatively, as shown in Table 2. No additional doses of idarucizumab were given. A mean 4F-PCC dose of 1071 IU (95% CI: 635 to 1507 IU) was given for VKA reversal, achieving INR reductions from a preoperative median of 2.13 to 1.87 postoperatively.

Transfusion rates varied between the groups (Table 3). In the DBG cohort, 55% (11/20) of patients required transfusions in contrast with 92.9% (13/14) in the VKA cohort and all patients (14/14) in the NA cohort. Regarding the total number of blood products used, statistically significant differences were found in DBG vs. VKAs (*p* < 0.001) and DBG vs. NA (*p* = 0.008) but not in VKAs vs. NA (*p* = 0.194). Despite a 55% transfusion rate in the DBG cohort, the median number of blood products used was relatively low (one unit, IQR 0–4), indicating that while more than half of the patients required a transfusion, the overall volume of transfusion needed per patient was significantly lower compared to the VKA cohort (median 13.5 units, IQR 4–19) and NA cohort (median seven units, IQR 2–15). This suggests that while transfusions were frequently needed in the DBG group, they were less extensive in terms of blood product volume compared to the other cohorts. Specifically, differences were found with PRBC use, particularly between DBG and VKAs (*p* = 0.001), while comparisons between DBG vs. NA, and VKAs vs. NA, were not significant (*p* = 0.073 and *p* = 0.088). A similar pattern was found in the case of FFP transfusions. Lastly, for POP transfusions, significant differences were found between DBG and VKAs (*p* = 0.009), between DBG and NA (*p* = 0.013), but not between VKAs and NA (*p* = 0.423). Two of the three individuals in the VKA group with mechanical mitral valves and INR targets between 2.5 and 3.5 actually had a preoperative INR below three but still required a high number of transfusions, with 25 and 28 blood products used.

The assessment of re-surgery requirements, 30-day survival rates, and ICU stay lengths are also described in Table 3. The occurrence of re-surgery due to bleeding did not show statistically significant differences across the groups (*p* = 0.259). Regarding the 30-day survival rate, each group experienced one death, but it was only attributed to bleeding in the VKA and NA group. The patient who passed away in the DBG cohort did so because of graft rejection. Finally, the analysis showed that the ICU stay duration was significantly shorter in the DBG group compared to both the VKA (*p* = 0.014) and NA groups (*p* = 0.009) but not between VKAs and NA (*p* = 0.376).

### 3.3. Cost Analysis

The analysis evaluates the expenses associated with using idarucizumab versus VKA reversal, as detailed in Table 4. The cost analysis, as detailed in Table 4, compares the financial aspects of two treatment strategies. Two 2.5 mg vials of idarucizumab were used for each patient, which at the point of this study totaled EUR 6356, whilst the median cost of VKA reversal was EUR 429. Patients on DBG showed a median transfusion cost of EUR 400, which was lower when compared to the median of EUR 2085 in the VKA cohort (*p* = 0.001). Additionally, as the DBG cohort stayed in the ICU for fewer days than the VKA cohort, the median ICU cost was EUR 10,087 and EUR 12,608, respectively (*p* = 0.02). Taking all these costs together, the difference between the two cohorts was quite similar, with medians of EUR 16,987 (IQI 13,745 to EUR 19,574) for the DBG and EUR 15,520 (IQI 13,357 to EUR 19,144) for the VKA cohort (*p* = 0.4). The sensitivity analysis (Table 4) revealed that ICU stay duration was a major driver for variability, with global costs ranging from a median cost difference of EUR 4344 lower in the DBG cohort in the best-case scenario to being EUR 4062 greater in the worst-case scenario.

## 4. Discussion

Our study offers new insights into anticoagulation reversal strategies prior to HT, evaluating the effectiveness of idarucizumab for patients on DBG compared to those undergoing VKA reversal. Reversal with idarucizumab was associated with reduced transfusion requirements and shorter ICU stays without impacting the rates of re-surgery due to bleeding or 30-day survival. As the first study to directly compare the outcomes of reversing DBG with idarucizumab against a VKA reversal, our findings highlight a potential benefit of choosing DBG for patients awaiting HT.

Many patients on HT waiting lists require anticoagulation for different indications and thus have a higher risk of severe perioperative hemorrhagic complications. VKAs are used to anticoagulate such patients whilst on the waiting list, as they can be reversed with the administration of vitamin K in combination with 4F-PCC or alternatively, low dose three-factor PCCs supplemented with FFP if 4F-PCC is not available [7]. The unpredictable availability of donor hearts coupled with the complex logistics of the transplantation procedure warrants an immediately accessible and reliable anticoagulation reversal strategy, as these circumstances frequently result in an extremely limited time frame for effective anticoagulant reversal [2]. Hemostasis after the administration of vitamin K plus 4F-PCC is usually achieved within approximately 1 h (±1 h) [8].

Alternatively, DOACs have increasingly replaced VKAs to prevent stroke and systemic embolic events in patients with NVAF, offering advantages such as treatment simplification, no need for monitoring, no dietary restrictions, and an overall better risk–benefit ratio [3,4]. However, the lack of a DOAC antidote limited its use in patients requiring emergency surgery until 2015, when idarucizumab was approved for DBG reversal based on the results of the RE-VERSE AD trial, which included patients on emergency surgery [9]. Idarucizumab is a humanized monoclonal antibody fragment that binds to free and thrombin-bound DBG, neutralizing its anticoagulant effects very effectively within 5 min [1,10,11]. All patients in the DBG cohort achieved hemostatic control post-idarucizumab exposure, evidenced by a sharp drop in the aPTT to a normal range, ruling out significant residual effects of DBG [9,11].

Idarucizumab is currently the only DOAC antidote with an indication in emergency surgery, as andexanet alfa, a factor Xa inhibitor, cannot be used in this setting because it interferes with heparinization [12]. Its use in cardiac surgery is endorsed in the setting of emergency surgery by the main cardiothoracic surgical guidelines [13,14,15,16], although available evidence in the setting of HT is sparse. The RE-VERSE AD trial full cohort analysis only included 37 patients with cardiovascular emergency procedures, none of them being HT. To the best of our knowledge, there are currently a total of four case reports [9,17,18,19], two case series [11,20], and a retrospective multicentric study [2] that report on the use of idarucizumab in HT, with promising results.

In our study, the DBG cohort was associated with a lower risk of being transfused in comparison with that of VKAs, with only 55% of patients in the DBG cohort requiring transfusions and a median of one blood product being used compared to 100% in the VKA cohort being transfused and a median of 13.5 blood products used (*p* < 0.001). No differences in the rates of re-surgery due to bleeding were noted, although there was one death related to massive bleeding in both the VKA and NA group. The ICU stay duration was slightly shorter in the DBG cohort compared to the VKA group, with a median of 6 days in the DBG group versus 7.5 days in the VKA group (*p* = 0.014). While HAS-BLED scores indicated low bleeding risk in both DBG and VKA cohorts, other factors we considered were not entirely balanced. Specifically, 14.2% of VKA patients underwent more complex surgeries with combined organ transplants—circumstances not observed in the DBG group—while the DBG cohort showed a slightly lower incidence of previous sternotomies (15.8% vs. 21.4%) and no use of anti-aggregation therapy in contrast to 14.3% in the VKA group. Additionally, the differences in renal function between the groups might have introduced some bias regarding the bleeding risk. Although HAS-BLED accounts for renal function, we acknowledge that even less marked differences may still contribute.

Unexpectedly, the NA cohort exhibited higher transfusion requirements and longer ICU stays, likely due to more prevalent bleeding risk factors, undermining its validity as a control group. For instance, a history of previous sternotomy was notably more prevalent (42.9%), as well as a higher use of anti-aggregation therapy (46.2%). Additionally, the frequency of HT performed during the course of ongoing hospitalizations was markedly higher in NA patients (42.9%) compared to the DBG group (5%) and the VKA group (0%). Typically, such patients are at advanced stages of disease and demonstrate clinical instability, which adds complexity to surgeries and elevates the risk of bleeding complications.

Regarding the effects on coagulation tests, all patients in the DBG cohort achieved hemostatic control post-idarucizumab exposure, evidenced by a drop in the aPTT from a median of 47 to 28 s. While aPTT is not the optimal method to assess DBG anticoagulant activity, it is a reliable indicator to rule out significant residual anticoagulant effects [1], as demonstrated in the RE-VERSE AD trial and mirrored by results from diluted thrombin time tests [9]. Van Keer et al.’s study also showed a decrease in both aPTT and DBG levels following idarucizumab administration [11]. Additionally, idarucizumab, which remains in the blood vessels, has been associated with a rebound in plasma DBG levels, necessitating additional half-doses in some cases [11]. This is particularly noted in patients with renal disease, where the redistribution of DBG from the extravascular to the vascular compartment occurs after the renal clearance of idarucizumab. This rebound typically begins 4 h post-administration, peaking at 12 h [21]. In our cohort, no additional idarucizumab doses were required, likely due to most patients having an eGFR above 50 mL/min/1.73 m^2^. Therefore, our findings should not be generalized to patients with lower renal function, as they may have a higher bleeding risk compared to VKA reversal. Future research should aim to address this specific patient group.

Our cost analysis revealed that reversing DBG with idarucizumab is on average EUR 6000 more expensive than reversing VKAs. However, this cost disparity is partly offset by the higher transfusion requirements in the VKA cohort and notably, the shorter ICU stays in the DBG cohort effectively balanced the overall expenses. Consequently, we observed no statistically significant difference in the overall costs between the two cohorts. The sensitivity analysis clearly demonstrates that ICU costs exert a profound influence on overall cost variability. In the DBG-low-VKA-high scenario, utilizing idarucizumab provided a cost advantage of EUR 4344. Conversely, in the DBG-high-VKA-low scenario, the use of VKAs resulted in an increased cost of EUR 4062. This analysis underscores the significant impact of ICU cost variability on global costs. Obtaining more precise ICU cost inputs could enable future research to achieve narrower confidence intervals, leading to more definitive cost projections in similar studies.

Our study suggests that reversing dabigatran with idarucizumab before heart transplantation reduces the need for transfusions and the length of ICU stays, without a statistically significant increase in costs compared to vitamin K antagonist reversal. However, several limitations warrant attention. The study’s retrospective design and small sample size are primary constraints. Despite incorporating all anticoagulated heart transplant patients available during the study period, the sample size remained limited, which is noteworthy given Spain’s high number of heart transplants and our center’s position among the top five in Spain for such procedures. The inherent scarcity of such specific patient populations contributed to the smaller and less homogeneous than desired sample size. Future multicentric controlled studies involving broader patient populations are needed to validate our findings.

## Figures and Tables

**Table 1:** Reversal agent, blood product, and our hospital's average daily ICU cost.

Item	Cost (EUR)
Reversal agents	
Phytonadione (Vitamin K) 10 mg/1 mL	9.37
4F-PCC (Octaplex^®^) 500 UI	210
4F-PCC (Octaplex^®^) 1.000 UI	420
Idarucizumab 2.5 g	3178
Blood products	
PRBC	145
FFP	60
POP	400
Hospital admission cost	
Daily Average ICU Stay ^a^	1681.11

Abbreviations: ICU, intensive care unit; FFP, fresh frozen plasma; POP, pool of platelets; PRBC, packed red blood cell; 4F-PCC, 4-factor prothrombin complex concentrate. ^a^ The cost per ICU day reflects the daily cost per patient in an already functioning ICU ward at our hospital. This amount includes imaging tests and laboratory services and represents an average operating cost. It does not include building or facility maintenance costs.

**Table 2:** Patient baseline, coagulation, and anticoagulant reversal profiles.

	DBG Cohort (n = 20)	VKA Cohort (n = 14)	NA (n = 14)
Basal Characteristics			
Age, median years (IQI)	54.7 (41.5–63.0)	53.2 (40.6–60.6)	51.7 (35–58.7)
Gender, % males	70	43	71.4
Underlying heart disease, %			
- Dilated cardiomyopathy (ischemic) - Dilated cardiomyopathy (non-ischemic) - Hypertrophic cardiomyopathy - Arrhythmogenic cardiomyopathy - Graft failure/disease - Congenital heart diseases (e.g., failed Fontan) - Other specific conditions (e.g., amyloidosis)	3 (15) 5 (30) 8 (40) 1 (5) 1 (5) 1 (5) 1 (5)	4 (28.6) 4 (28.6) 1 (7.1) 0 (0) 1 (7.1) 2 (14.3) 2 (14.3)	3 (21.4) 2 (14.3) 1 (7.1) 2 (14.3) 1 (7.1) 1 (7.1) 4 (28.6)
eGFR, median mL/min/1.73 m^2^, (IQI)	>60	53	>60
	(55–>60)	(37–59)	(48–>60)
Bleeding Risk Characteristics			
HAS-BLED score, median (IQI)	0 (0)	0 (0–1)	N/A
Only or combined HT, n (%)			
Heart only Cardiorenal Cardiohepatic	20 (100) 0 (0) 0 (0)	12 (85.7) 1 (7.1) 1 (7.1)	13 (92.9) 0 (0) 1 (7.1)
Previous sternotomy n (%)	3 (15.8)	3 (21.4)	6 (42.9)
Anti-aggregation n (%)	0 (0)	2 (14.3)	6 (46.2)
HT during index admission, n (%)	1 (5)	0 (0)	6 (42.9)
CPB time, median minutes (IQI)	135 (124–150)	185 (133–208)	157 (145–171)
Anticoagulant, Coagulation, and Reversal Characteristics			
Anticoagulant indication			
Non-valvular AF, atrial flutter, n (%) Mechanical mitral valve, n (%)	20 (100) 0	12 (85.7) 2 (14.3)	N/A N/A
CHA_2_DS_2_-VASc score, median (IQI)	2 (1–3)	2 (2–3)	N/A
DBG dose			
150 mg twice daily, n (%) 110 mg twice daily, n (%)	15 (75) 5 (25)	N/A N/A	N/A N/A
Preoperative aPTT, median seconds (IQI)	47.4 (40.7–51.4)	39.95 (38.9–44.1)	31.5 (28.5–43.3)
Postoperative aPTT, median seconds (IQI)	28.2 (25.4–32)	31.7 (29.2–35.5)	32.4 (28.4–34.0)
VKA drug			
Acenocoumarol Warfarin	N/A N/A	13 (92.9) 1 (7.1)	N/A N/A
INR target	N/A		N/A
2 to 3, n (%) 2.5 to 3.5, n (%)		12 (85.7) 2 (14.3)	
Preoperative INR, median (IQI)	1.18 (1.10–1.29)	2.13 (1.77–2.71)	1.16 (1.06–1.32)
Postoperative INR, median (IQI)	1.32 (1.2–1.47)	1.87 (1.74–2.19)	1.40 ^a^ (1.29–1.55)
Reversal agent dose			
Idarucizumab, g	2 × 2.5	N/A	N/A
Vitamin K, mg	N/A	10	N/A
4F-PCC, mean IU (95% CI)	N/A	1071 (635–1507)	N/A
Preoperative Hb, median g/gL (IQI)	14.8 (12.9–16.1)	14.5 (13.3–15.6)	13.2 (9.9–16.9)
Postoperative Hb, median g/gL (IQI)	10.2 (9.8–11.7)	9.9 (8.6–11)	9.8 a (9.2–10.8)

^a^: One patient from the NA cohort passed away during surgery. Thus, the postoperative INR and Hb results were calculated with n = 13. Abbreviations: aPTT, activated partial thromboplastin time; CPB, cardiopulmonary bypass; CI, confidence interval; DBG, dabigatran; eGFR, estimated glomerular filtration rate; Hb, hemoglobulin; INR, international normalized ratio; IQI, interquartile interval; N/A, Not Applicable; VKA, vitamin K antagonist; 4F-PCC, 4-factor PCC.

**Table 3:** Clinical outcome analysis of DBG and VKA reversal in HT.

	DBG Cohort (n = 19)	VKA Cohort (n = 14)	NA Cohort (n = 14 ^b^)	Kruskal–Wallis Chi-Square and *p*-Value	Dunn Post Hoc Comparisons with Holm–Bonferroni-Adjusted *p*-Value
Total Number of Perioperative Blood Products, Median Units (IQI) ^a^	1 (0–4)	13.5 (4–19)	7 (2–15)	χ = 14.78 *p* < 0.001	DBG vs. VKA: *p* < 0.001 DBG vs. NA: *p* = 0.008 VKA vs. NA: *p* = 0.194
Perioperative PRBC Transfusions, Median Units (IQI) ^a^	0 (0–2)	5 (1–10)	4 (1–5)	χ = 17.61 *p* < 0.001	DBG vs. VKA: *p* = 0.001 DBG vs. NA: *p* = 0.073 VKA vs. NA: *p* = 0.088
Perioperative FFP Transfusions, Median Units (IQI) ^a^	0 (0–0.5)	4.5 (0–10)	2 (0–4)	χ = 11.38 *p* = 0.003	DBG vs. VKA: *p* = 0.001 DBG vs. NA: *p* = 0.073 VKA vs. NA: *p* = 0.088
Perioperative POP Transfusions, Median Units (IQI) ^a^	0 (0–1)	2.5 (0–4)	1 (1–2)	χ = 9.94 *p* = 0.007	DBG vs. VKA: *p* = 0.009 DBG vs. NA: *p* = 0.013 VKA vs. NA: *p* = 0.423
ICU Stay, Days (IQI)	6 (4–7)	7.5 (7–9)	8 (7–17)	χ = 9.80 *p* = 0.008	DBG vs. VKA: *p* = 0.014 DBG vs. NA: *p* = 0.009 VKA vs. NA: *p* = 0.376
HAS-BLED Score, Median (IQI)	0 (0)	0 (0–1)	1.5 (0.5–2.5)	χ = 8.15 *p* = 0.017	DBG vs. VKA: *p* = 0.230 DBG vs. NA: *p* = 0.007 VKA vs. NA: *p* = 0.062
CPB Time, Median Minutes (IQI)	135 (124–150)	185 (133–208)	157 (145–171)	χ = 5.04 *p* = 0.080	N/A
				Pearson’s Chi-Square and *p*-Value	Pearson’s Chi-Square Post Hoc Comparisons with Holm–Bonferroni-Adjusted *p*-value
Number of Patients Requiring Any Transfusion, n (%)	11 (55)	13 (92.9)	14 (100)	χ = 11.90 *p* = 0.003	DBG vs. VKA: *p* = 0.034 DBG vs. NA: *p* = 0.005 VKA vs. NA: *p* = 0.978
Re-Surgery due to Bleeding, n (%) ^a^	1 (5)	3 (21.4)	1 (12.5)	χ = 2.70 *p* = 0.259	N/A
30-Day Survival Rate, n (%)	18 (94.7)	13 (92.9)	13 (92.9)	χ = 0.09 *p* = 0.955	N/A

^a^ Data on transfusions and re-surgeries due to bleeding cover the period from surgery to 48 h post-surgery. ^b^ One patient experienced severe bleeding during surgery, leading to a decision against further intervention or transfusion. To avoid underestimating the transfusion need, this patient was considered as transfused. However, for the rest of the postoperative outcome calculations (i.e., number of blood products transfused, ICU stay length, and re-surgery due to bleeding), the patient was excluded (n = 13) to prevent distorting these results. Abbreviations: DBG, dabigatran; FFP, fresh frozen plasma; ICU, intensive care unit; IQI, interquartile interval; POP, pool of platelets; PRBC, packed red blood cell; VKA, vitamin K antagonist.

**Table 4:** Cost analysis comparing the reversal, transfusion, and ICU expenditure of patients from the dabigatran and vitamin K antagonist cohorts.

	DBG Cohort (n = 20)	VKA Cohort (n = 14)	*p*-Value
Reversal Cost per Patient, Median EUR (IQI)	6356 (6356)	429 (219–1059)	N/A
Transfusion Cost per Patient, Median EUR (IQI)	400 (0–690)	2085 (435–3795)	0.001
ICU Cost per Patient, Median EUR (IQI)	10,087 (6724–11,768)	12,608 (11,768–15,130)	0.02
Global Cost, Median EUR (IQI)	16,988 (13,745–19,574)	15,520 (13,357–19,144)	0.4

Abbreviations: DBG, dabigatran; ICU, intensive care unit; IQI, interquartile interval; VKA, vitamin K antagonist.

## Data Availability

Data are contained within the article.
